# Certified reference materials for GMO analysis—more than 25 years of GMO CRM production at EC JRC

**DOI:** 10.1007/s00216-024-05713-y

**Published:** 2025-01-23

**Authors:** Stefanie Trapmann, Thomas P. J. Linsinger, Robert Koeber

**Affiliations:** European Commission, Joint Research Centre, Directorate F – Health and Food, Geel, Belgium

**Keywords:** Certified reference material (CRM), Genetically modified organism (GMO), ISO 17034, ISO/IEC 17025

## Abstract

Certified reference materials (CRMs) play a crucial role in ensuring the quality of analytical measurements. Particularly, the EU labelling legislation on genetically modified organisms (GMOs) in food and feed products explicitly requires CRMs for its implementation. The CRMs are used to calibrate and control the quantitative real-time polymerase chain reaction (qPCR) method and support official control laboratories, such as National Reference Laboratories (NRLs), in maintaining their ISO/IEC 17025 accreditation. The Joint Research Centre of the European Commission (EC JRC) is specialized in the production of reference materials and has been producing GMO CRMs since 1998. Together with a validated event-specific qPCR method, these GMO CRMs form the EU reference system for the quantification of EU-authorized GMO events in food and feed products and ensure a common GMO labelling threshold. This article gives a retrospective view on the more than 25 years of GMO CRM production at JRC. It describes requirements for GMO CRMs in view of an EU market authorization under (EC) No. 1829/2003. The evolution and major improvements of the production of GMO CRMs at JRC are summarized as well as the current understanding of the EU’s GMO reference system for GMO quantification and its impact on commutability. It provides insights into GMO CRM sales and their worldwide distribution. This information may be useful for policymakers and researchers in understanding the current EU GMO measurement landscape and to anticipate possible future demands related to GMO events based on new genomic techniques (NGTs).

## Introduction

Certified reference materials (CRMs) are essential tools for the quality assurance of analytical measurements. Genetically modified organisms (GMOs) need to be authorized under (EC) No 1829/2003 [1] for the EU market and need to be labelled. The quantification of GMOs in food and feed products to implement EU labelling legislation requires CRMs to calibrate the quantitative real-time polymerase chain reaction (qPCR). Additionally, these GMO CRMs are used to verify the correct application of the quantification method and support official control laboratories, such as the (EU) 2017/625 National Reference Laboratories (NRLs) [2], to maintain their legally required ISO/IEC 17025 [3] accreditation.

The Joint Research Centre of the European Commission (EC JRC) is specialized in the production of reference materials and released in 1998 the first GMO CRMs. In the following years, further experience and expertise were gained and the production of GMO CRMs was improved, on the one hand ensuring the suitability of the CRMs for EU’s legal GMO framework and on the other hand guaranteeing an efficient production time which is not delaying the authorization.

The event-specific qPCR method proposed by the biotech company and validated by the European Union Reference Laboratory for Genetically Modified Food and Feed (EURL GMFF) [4] sets up the EU reference system for quantification of EU authorized GMO events in food and feed products together with the GMO CRM which is mentioned in the implementation decision of the EU market authorization.

## EU market authorization process and related requirements for official GMO CRMs

Regulation (EC) No 1829/2003 stipulates that the biotech company requesting the authorization of a GMO event as food and feed product for the EU market needs to ensure the accessibility to a reference material (RM) [1]. Further building blocks of the GMO legislation clarify that the RM needs to be a certified reference material (CRM) [5, 6] produced by an accredited reference material producer (RMP) [5]. EU legislation laying down requirements with respect to GMO RMs and details about the relevant text parts are given in Table 1.

**Table 1:** Current EU legislation laying down requirements with respect to GMO RMs

Legislation (in chronological order)	Quotation of relevant legal text parts
Regulation (EC) No 1829/2003 on genetically modified food and feed [1]	Chapter II/III Genetically modified food/feed Article 5 and 17 Application for authorisation (3) The application shall be accompanied by the following: (j) …. **information as to the place where the reference material can be accessed** Article 30 Confidentiality (5) The use of the detection methods and the reproduction of the reference materials, provided under Article 5(3) and 17(3) for the purpose of applying this Regulation to the GMOs, food or feed to which an application refers, **shall not be restricted by the exercise of intellectual property rights or otherwise.**
Regulation (EC) No 1830/2003 concerning the traceability and labelling of GMOs [7]	Article 7 Amendment of Directive 2001/18/EC Article 21 (3) For products intended for direct processing, paragraph 1 shall not apply to **traces of authorised GMOs in a proportion no higher** **than 0,9 %** or lower thresholds …. **provided that these traces are adventitious or technically unavoidable**
Regulation (EC) No 641/2004 on detailed rules for the implementation of Regulation (EC) No 1829/2003 [8]	Annex II Reference materials The reference material as referred to in Articles 5(3)(j) and 17(3)(j) of Regulation (EC) No 1829/2003 shall be produced in accordance with internationally accepted technical provisions such as ISO Guides 30 to 34* (and more particularly ISO Guide 34*, specifying the general requirements for the competence of reference material producers). The reference material shall be **preferably certified** and, if such is the case, certification shall be done in accordance with ISO Guide 35*. For verification and value assignment, a method that has been properly validated (see ISO/IEC 17025:2017, Section 5.4.5) shall be used. **Uncertainties have to be estimated according to GUM** (ISO Guide to the Expression of Uncertainty in Measurement: GUM). *Further details about RM container, homogeneity and stability testing, batch characterisation, final storage, establishment of a certificate for CRMs are given.*
Commission Recommendation (EC) 787/2004 on technical guidance for sampling and detection of genetically modified organisms and material produced from genetically modified organisms as or in products in the context of Regulation (EC) No 1830/2003 [9]	V point 4 Analytical testing If a positive result is obtained, **specific methods for a genetic construct and/or transformation event** should be carried out. The results of quantitative analysis should be expressed as the **percentage of GM-DNA copy numbers** in relation to target taxon specific DNA copy numbers calculated in terms of haploid genomes. *This recommendation was de-facto repealed by Regulation (EU) No 619/2011, which specified that percentages are understood as mass fractions*
Regulation (EU) No 619/2011 on the official control of feed as regards presence of genetically modified material for which an authorisation is pending or expired [6]	Article 3 Certified reference material (1) **Certified reference material must be available to Member States and any third party**. (2) Certified reference material shall be produced and certified in accordance with ISO Guides 30 to 35*. (3) The information accompanying the certified reference material shall include **information on the breeding of the plant which has been used for the production of the certified reference material and on the zygosity of the insert(s).** The **certified value of the GMO content shall be given in mass fraction** and, where available, in copy number per haploid genome equivalent.
Commission Implementing Regulation (EU) No 503/2013 on applications for authorisation of genetically modified food and feed in accordance with Regulation (EC) No 1829/2003 [5]	Annex III …. Requirements for ….the certified reference material (4) Certified reference materials The **certified reference material** shall be produced under ISO Guide 34* (General requirements for the competence of reference material producers) by a producer accredited to ISO Guide 34*. *Further detailed RM requirements are given in part 4.*
Regulation (EU) 2017/625 on official controls for food and feed law [2]	Article 37 Designation of official laboratories (4) The competent authorities may only designate as an official laboratory a laboratory which … (e) **operates in accordance with the standard EN ISO/IEC 17025 and is accredited in accordance with that standard** by a national accreditation body operating in accordance with Regulation (EC) No 765/2008.

*ISO Guide 31 to 35 were in the meantime replaced by ISO 33401:2024, ISO 33403:2024, ISO 33405:2024, and ISO 17034:2016

**Fig. 1:**
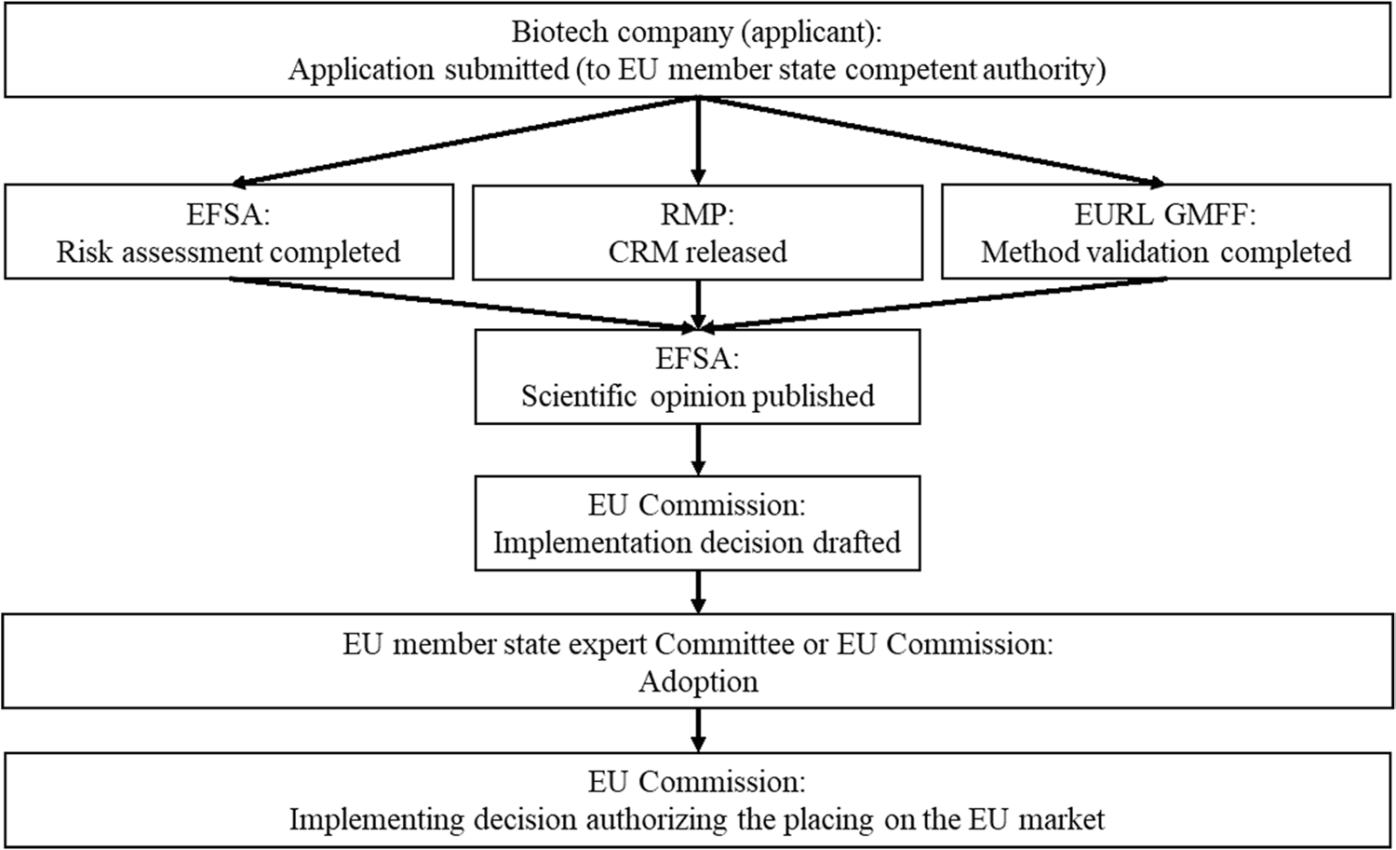
Simplified scheme of a successful GMO authorization for marketing of food and feed and derived products under (EC) No 1829/2003. EU market authorizations are granted for a maximum of 10 years and are renewable

For an EU market authorization of a given GMO event, the biotech company (referred to as applicant in (EC) No 1829/2003) notifies a national competent authority within the EU about its intention to place a GMO on the EU market. This triggers that a risk assessment is carried out by the European Food and Safety Authority (EFSA). In parallel, the applicant provides a detection method to the EURL GMFF for validation and contacts an RMP for the CRM production. The outcome of the risk assessment, the information about the accessibility of the GMO CRM, and a published, validated method form the three basic elements required for an authorization (see Fig. 1). In the EU, a GMO event may be authorized as food or feed product for a renewable period of 10 years, provided that the quantification method and GMO CRM remain accessible. In the context of this article, the GMO CRM mentioned in the implementation decision authorizing an GMO event for use in food and feed products is referred to as ‘official CRM’.

More detailed information about the EU authorization system can be found on the websites of the EC Directorate-General for Health and Food Safety (DG SANTE) [10].

## GMO CRMs production at JRC

The first GMO CRMs were produced and released in 1998 for the detection and quantification of GTS 40-3-2 soybean and Bt176 maize. These two sets of GMO CRMs were the product of a collaboration between the EC JRC Institute for Reference Materials and Measurements (IRMM, Geel, BE; now JRC, Directorate F – Health and Food) and Fluka (Buchs, CH, now part of Merck KgA, DE).

The need to regulate and monitor GMOs arose as a consequence of the increasing cultivation of GMOs worldwide. The EU started to establish an extensive legal framework for GMOs in early 1990, which was amended between 2000 and 2003, leading to an updated EU legal framework in 2003 [11]. Prior to 2003 and the specific GMO regulations (EC) No 1829/2003 and (EU) No 1830/2011, access to raw materials, such as seed materials, protected by intellectual property rights (IPR) was challenging. In these early years, laboratories depended on negotiating bilateral agreements with the biotech companies to obtain a material for calibration. For this reason, JRC’s first GMO CRMs were produced using harvested grain materials as raw materials instead of seeds. In the meantime, these CRMs have all been replaced by seed-based CRMs.

Regulations (EC) No 1829/2003 and (EC) No 1830/2003 request the labelling of food and feed products containing authorized GMO products, enabling EU citizens to choose between GMO-containing food and food not containing GMOs. The labelling threshold created the need for analytical laboratories to quantify GMOs in food and feed products precisely. Article 30 of (EC) No 1829/2003 not only did help RMPs to negotiate material transfer agreements (MTAs) for seed materials with the biotech company owning the IPRs but also guaranteed that the RMP has access to the quantification method, intended to be validated by EURL GMFF (initially referred to as Community Reference Laboratory for GMOs (CRL GMO)).

In 2004, Regulation (EC) No 641/2004 [8] and Recommendation (EC) 787/2004 [9] requested the quantification of the GMO event by PCR. As a consequence, the intended use of the GMO CRMs produced by JRC changed from a broader use for DNA and protein-based GMO quantification methods (e.g. IRMM-413, released in 2001) to an intended use limited to DNA-based applications.

From 2007 onwards, all GMO CRM certificates provide basic information about the breeding process and the zygosity status of the GMO seeds used for CRM production. This entails whether the seeds are homo- or heterozygous/hemizygous and from which parent (female or male) the GMO event was crossed into the GM seed line. This information is provided as additional information obtained from the biotech company, as it is not verified during the certification process.

ISO Guide 34 and its replacement ISO 17034:2016 [12] list the general requirements for RMPs. Regulation (EU) No 503/2013 restricted the production of the required official GMO CRMs to RMPs accredited to ISO Guide 34. JRC became accredited in 2004 for its GMO CRM production [13]. To further safeguard the quality of the official GMO CRMs, DG SANTE asked the EURL GMFF in 2019 to systematically check for the availability and appropriateness of the official GMO CRM when dealing with applications submitted under (EU) No 503/2013 and for renewal applications. Naturally, this includes checking the GMO CRMs produced by EC JRC.

From the beginning, the JRC opted to produce and provide sets of CRMs with different GMO mass fractions (m/m), allowing laboratories to use these mixtures directly for establishing calibration curves. In addition, the CRM with the mass fraction close to the implementation threshold (0.9% [7] or 0.1% [6]) could be used additionally for a partial quality control. All percentages given in this article are mass fractions expressed in per cent, unless stated otherwise. This setup has the disadvantage that the CRM used for quality control is not independent from the CRM used for calibration and a bias in all of them would remain undetected. Using one CRM of the sets produced from the same raw material for calibration, nevertheless, allows to check the general functioning of the quantification method. The setup with plasmid calibrants (see below) would have solved this issue, but turned out unfeasible for other reasons.

Initially, a nominal 0% and varying levels with mass fraction between 0.1 and 10% were released per set of GMO CRM produced at JRC. At that time, the nominal 1% level was envisaged as EU food labelling threshold, and a lower mass fraction was selected to facilitate the verification of the performance criteria of the quantification method. If no nominal 100% level was released, a nominal 10% level was added, as it supports higher GMO thresholds established in some countries outside the EU.

The later established threshold of 0.1% for GMO events with a pending or expired authorization in feed as laid down in (EU) No 619/2011, asked for the systematic production of 0.1% GMO CRMs. The production and certification of nominal levels of 0, 0.1, 1, and 10% became the preferred way from 2005 onwards (see Table 2). Using tenfold dilution steps between the different levels ensured material preparation, which also meant that the amount of GMO seeds requested by JRC from the biotech companies could be lowered.

**Table 2:** Set of GMO CRMs released by EC JRC between 1998 and 2024 and their authorization status in 2024 under (EC) No 1829/2003 [15] with ‘authorized’ (listed in the EU Register of authorized GMOs), ‘pending’ (active (EC) No 1829/2003 authorization request, benefitting from (EU) No 619/2011), ‘expired’ (no active (EC) No 1829/2003 authorization request, benefitting from (EU) No 619/2011) and ‘unauthorized’ (no active (EC) No 1829/2003 authorization request (yet), not (yet) benefitting from (EU) No 619/2011). Besides the GMO event name used on the CRM unit, the unique identifier given to the GMO event by the Organisation for Economic Co-operation and Development (OECD) is provided

GMO event (OCED unique identifier) and species	Code of the CRM set	(EC) No 1829/2003 authorization status (and, if, official CRM)	Nominal mass fraction levels made available [% (m/m)] (and, if applicable, plasmid calibrant)	Release year (and, if deviating, CRM code of first release)
GTS 40-3-2 (MON-04032-6) soybean	ERM-BF410	authorized (official CRM for (EC) No 1829/2003)	0, 0.1, 1, 10, 100	1998 (IRMM-410) 2000 (IRMM-410R) 2002 (IRMM-410S*) 2008 (ERM-BF410k) 2016 (ERM-BF410dn, gn) 2017 (ERM-BF410p)
Bt176 (SYN-EV176-9) maize	ERM-BF411	expired (official CRM for (EU) No 619/2011)	0, 0.1, 0.5, 1, 2, 100	1999 (IRMM-411) 2003 (IRMM-411R*) 2024 (ERM-BF411g)
Bt11 (SYN-BTØ11-1) maize	ERM-BF412	authorized (official CRM for (EC) No 1829/2003)	0, 0.1, 1, 10, 100	2000 (IRMM-412) 2003 (IRMM-412R*)
MON 810 (MON-ØØ810-6) maize	ERM-BF413 ERM-AD413	authorized (official CRM for (EC) No 1829/2003)	0, 0.5, 2, 10 pDNA	2001 (IRMM-413*) 2007 (ERM-AD413) 2009 (ERM-BF413k)
GA21 (MON-ØØØ21-9) maize	ERM-BF414	authorized	0, 0.1, 0.5, 1, 2, 5	2004 (IRMM-414*)
NK603 (MON-ØØ6Ø3–6) maize	ERM-BF415 ERM-AD415	authorized (official CRM for (EC) No 1829/2003)	0, 0.1, 0.5, 1, 2, 5, 100 pDNA	2004 2011 (ERM-AD415) 2024 (ERM-BF415g)
MON 863 (MON-00863-5) maize	ERM-BF416	expired (official CRM for (EU) No 619/2011)	0, 0.1, 1, 10	2005
1507 (DAS-Ø15Ø7-1) maize	ERM-BF418	authorized (official CRM for (EC) No 1829/2003)	0, 0.1, 1, 10	2005
H7-1 (KM-ØØØH71-4) sugar beet	ERM-BF419	authorized (official CRM for (EC) No 1829/2003)	0, 100	2006
3272 (SYN-E3272-5) maize	ERM-BF420	pending	0, 1, 10	2007
EH92-527-1 (EH92-527-1) potato	ERM-BF421	unauthorized (authorized for cultivation, industrial use)	0, 100	2006
281-24-236x3006-210-23 (DAS-24236- 5 x DAS-21Ø23-5) cotton	ERM-BF422	authorized (official CRM for (EC) No 1829/2003)	0, 1, 10, 100	2006
MIR604 (SYN-IR6Ø4–5) maize	ERM-BF423	authorized (official CRM for (EC) No 1829/2003)	0, 0.1, 1, 10	2006
59122 (DAS-59122-7) maize	ERM-BF424	authorized (official CRM for (EC) No 1829/2003)	0, 0.1, 1, 10	2006
356043 (DP-356Ø43-5) soybean	ERM-BF425 ERM-AD425	unauthorized	0, 0.1, 1, 10 pDNA	2007 2011 (ERM-AD425)
305423 (DP-3Ø5423-1) soybean	ERM-BF426	authorized (official CRM for (EC) No 1829/2003)	0, 0.5, 1. 10	2007
98140 (DP-Ø9814Ø−6) maize	ERM-BF427 ERM-AD427	unauthorized	0, 0.5, 2, 10 pDNA	2009 2011 (ERM-AD427)
GHB119 (BCS-GHØØ5–8) cotton	ERM-BF428	authorized (official CRM for (EC) No 1829/2003)	0, 1, 10	2009
T304-40 (BCS-GHØØ4–7) cotton	ERM-BF429	authorized (official CRM for (EC) No. 1829/2003)	0, 1, 10	2010
AM04-1020 (BPS-A1Ø2Ø−5) potato	ERM-BF430	unauthorized (authorized for cultivation, industrial use)	0, GMO certified for identity	2011
AV43-6-G7 (BPS-A1Ø2Ø−5) potato	ERM-BF431	unauthorized	0, GMO certified for identity	2012
DAS-68416-4 (DAS-68416-4) soybean	ERM-BF432	authorized (official CRM for (EC) No 1829/2003)	0, 0.5, 1. 10	2012
DAS-40278-9 (DAS-4Ø278-9) maize	ERM-BF433	authorized (official CRM for (EC) No 1829/2003)	0, 0.5, 1. 10	2012
73496 (DP-Ø73496-4) rapeseed	ERM-BF434	authorized (official CRM for (EC) No 1829/2003)	0, 0.1, 1, 10, 100	2013
PH05-026–0048 (BPS-PHØ48-1) potato	ERM-BF435	unauthorized (authorized for cultivation, industrial use)	0, 100 (certified for identity)	2014
DAS-44406-6 (DAS-444Ø6-6) soybean	ERM-BF436	authorized (official CRM for (EC) No 1829/2003)	0, 0.1, 1, 10, 100	2013
DAS-81419-2 (DAS-81419-2) soybean	ERM-BF437	authorized (official CRM for (EC) No 1829/2003)	0, 0.1, 1, 10, 100	2014
VCO-01981-5 (VCO-Ø1981-5) Maize	ERM-BF438	unauthorized	0, 0.1, 1, 10, 100	2015
DP-004114-3 (DP-ØØ4114-3) maize	ERM-BF439	authorized (official CRM for (EC) No 1829/2003)	0, 0.1, 1, 10, 100	2015
DAS-81910-7 (DAS-8191Ø−7) cotton	ERM-BF440	pending (official CRM for (EU) No 619/2011)	0, 0.1, 1, 10, 100	2018
DBN-09004-6 (DBN-Ø9ØØ4–6) soybean	ERM-BF441	unauthorized	0, 0.1, 1, 10, 100	2023
GHB811 (BCS-GH811-4) cotton	ERM-BF442	authorized (official CRM for (EC) No 1829/2003)	0, 0.1, 1, 10, 100	2021
GMB151 (BCS-GM151-6) soybean	ERM-BF443	authorized (official CRM for (EC) No 1829/2003)	0, 0.1, 1, 10, 100	2021
DP202216 (DP-2Ø2216-6) maize	ERM-BF444	authorized (official CRM for (EC) No 1829/2003)	0, 0.1, 1, 10, 100	2023 (ERM-BF444a,b) 2024 (ERM-BF444c,d,e)
DP23211 (DP-Ø23211-2) maize	ERM-BF445	authorized (official CRM for (EC) No 1829/2003)	0, 100	2022
MIR162 (SYN-IR162-4) maize	ERM-BF446	authorized (official CRM for (EC) No 1829/2003)	0, 0.1, 1, 10, 100	2022

*The same batch of materials were released under the ERM brand in 2004

The production of a 0.1% CRM could turn out impossible for various reasons: the qPCR method did not have the required limit of detection (LOD), the method repeatability at this mass fraction level was insufficient, or the powder was for technical reasons not ground fine enough, or the non-GMO seeds received contained too high traces of the GMO event. In these cases, the nominal 0.1% CRM was replaced by a nominal 0.5%.

From 2013 onwards, the sets of GMO CRM were systematically amended with a nominal 100% (see Table 2). Releasing a pure GMO CRM became also useful in conjunction with the conversion factor (CF_CRM_) established by the EURL GMFF [14] for digital PCR applications from 2018 onwards. Digital PCR (dPCR) started to be used by some NRLs and control laboratories, requiring a common factor to convert the dPCR results expressed in DNA copy number ratios into GMO mass fractions. Besides this, a nominal 100% will be specifically useful for GMO events for which authorization was retracted or for which authorization was not prolonged.

All CRMs of a set produced at JRC are generally certified for their mass fraction of GMO, following EU legislation demanding to express the GMO content as mass fraction. For implementation of the current EU GMO framework, the GMO CRMs need to be used in conjunction with the EURL GMFF validated qPCR method.

EU legislation authorizes individual GMO events, requiring the use of event-specific qPCR methods and excluding screening methods from the final step to quantify the GMO mass fraction. At the same time, many biotech companies restrict the use of the GMO CRM in their MTA with the JRC to calibration and quality control of methods for the identification and quantification of GMO in food and feed. Therefore, the CRM certificates specify that GMO CRMs may not be used for other applications such as method development or genome sequencing.

With the authorization requests for stacked events (GMOs that contain more than one modification, e.g. created by cross-breeding of two GMOs), the technical question arose how to quantify them. As no discrimination of single events from stacked events can be made in processed food products (in contrast to seeds or, e.g., maize cobs) in routine control measurements, JRC maintained its approach to produce single event GMO CRMs, unless the single events of a stacked event did not yet undergo authorization in the EU (e.g. ERM-BF422 stacked cotton 281-24-236 x 3006-210-23). The only exception is ERM-BF417 (stacked event MON863 x MON810 maize [16]) which was originally released to facilitate research on the development of methods discriminating between single and stacked events.

## Major improvement of GMO CRM production at JRC

**Fig. 2:**
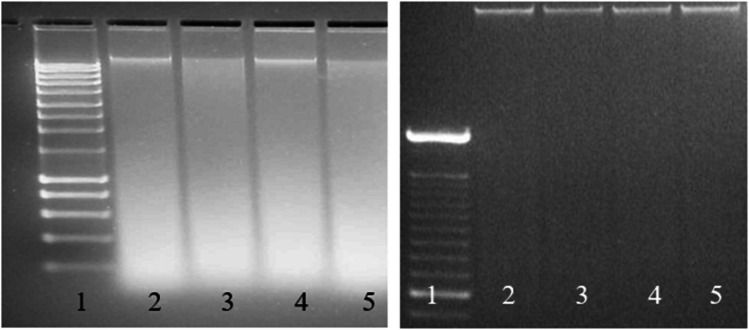
Gel electrophoresis of DNA extracted from maize seed powder after different processing steps of wet-mixing (left, lane 1: 100-bp DNA ladder, lane 2: first grinding and vacuum drying, lane 3: after additional milling, lane 4: after cryo-grinding, lane 5: after second grinding) and dry-mixing (right, lane 1: 100-bp DNA ladder, lane 2: propeller mixing with Tris-buffer, lane 3: propeller mixing with EDTA-buffer, lane 4: Turrax mixing, lane 5: propeller mixing without additives)

Obtaining a homogenous mixture of GMO and non-GMO powders during material processing is technically challenging. At first, a wet-mixing technique was used. A thorough mixing process was ensured through the addition of water to the powders, creating a slurry. The added water was removed by a time- and energy-intensive freeze-drying process. While resulting in superior homogeneity, wet mixing also has disadvantages. For one, the creation of a slurry partly pre-extracts the DNA which is then deposited on the outside of the particles and might therefore be extracted more easily from the CRM than from a food or feed product. This effect might make the results on CRMs less representative and might also introduce an analytical bias. In addition, wet-mixing led to DNA fragmentation as shown in Fig. 2. This did not negatively impact qPCR methods, which target even shorter DNA fragments, but hampered stability monitoring by gel electrophoresis, for which independent calibrants (DNA molecular-weight size markers, such as a 100-bp DNA ladder) are available as opposed to qPCR, that requires the very same CRM for calibration whose stability needs to be monitored. Dry-mixing techniques that do not degrade the DNA (see Fig. 2) were therefore developed and validated. For the validation of the dry-mixing technique, it needed to be proven that the resulting mixed powders are sufficiently homogenous. A dedicated study was performed mixing gold-spiked non-GM maize powder with non-spiked GM powder. The gold-spiking levels of the resulting mixtures were determined with the help of neutron activation analysis (NAA) measurements and confirmed by ELISA (enzyme-linked immunosorbent assay), targeting specifically the GM protein [17]. In the meantime, all wet-mixed GMO CRMs have been replaced by dry-mixed CRMs.

Among the general requirements for RMPs published in 2000 and 2009 (under ISO Guide 34) and updated in 2016 (under ISO 17034 [12]) is the requirement to assess long-term stability. Implementing Regulation (EU) No 503/2013 also specifically lists this as requirement for official GMO CRMs. To achieve a low uncertainty contribution for the potential instability of the certified value of a CRM, ideally measurement data covering several years are used. After collection of stability data for most important species (maize, soybeans, sugar beet, rapeseed, cotton, and potato) over several years, the JRC decided to apply an alternative approach by using these stability data to calculate estimates for uncertainties due to long-term instability in contrast to dedicated, individual stability studies. This allowed to shorten the CRM production time and ensures that the uncertainty of stability contributes only marginally to the overall uncertainty. However, this approach is only valid if the same processing techniques were applied to GMO events of comparable nature. In order to gain evidence that a new candidate GMO CRM behaves in the same way as an earlier produced GMO CRM, data obtained in short-term stability studies are compared. If they show the same behaviour, it is reasonable to transfer long-term stability data from earlier GMO CRMs to the new GMO CRM. For GMO CRMs certified for mass fractions, the JRC therefore has adopted the following strategy for the stability monitoring: Of each species (maize, soybean etc.), one ‘lead’ CRM is tested half-yearly until it is 5 years old; from then onwards, it is tested yearly. The other CRMs of that matrix are tested yearly for their first 5 years, then every 2 years.

For GMO CRMs intended for qualitative analysis (e.g. modified sugar beet, likely to be tested at the plant level rather than as food product and (starch) modified potatoes for industrial applications), a less stringent regime is necessary, as only a presence/absence result is required. These materials are subjected to additional stability monitoring every 3 years. In addition, only the GMO CRM is tested or its certified value, as it is impossible that DNA degradation in the non-GMO CRM will lead to the creation of GMO DNA (of exactly the specified event).

Release of (EC) No. 1829/2003 and access to raw materials of seed quality were an essential improvement [17]. Seeds have a high purity and known zygosity status. Especially for heterozygous species such as maize, this has a major impact. The purity of the GMO seed batches received from the biotech company was measured at JRC using 200 seeds. This did not need to be a qPCR technique as presence/absence testing for the GMO event in question is sufficient. This was, for example, done for CRMs for potato and sugar beet, where each individual tuber was tested for carrying the modification by staining with an aqueous iodine/iodide solution (EH92-527-1, AM04-1020, and AV43-6-G7 potato) or qPCR (PH05-026–0048 potato, H7-1 sugar beet). Obviously, this strategy is only feasible for relatively large items and can in practice not be implemented for small seeds like soybean or maize. The obtained data are used to establish a 95% confidence level for the minimum purity. In order to ensure that the GMO status of the individual seeds was correctly classified, seeds were decontaminated to remove attached dust particles, allowed to germinate and testing of the leaves of the young plants. In a further refinement of this approach, JRC’s purity measurements were combined with the purity data obtained by the seed producer, provided that they were obtained in a laboratory adhering to ISO/IEC 17025 requirements.

In the beginning, the same approach was used to verify the purity of the non-GMO seed batch. With the general improvement of qPCR, its accuracy and specifically with improved (lowered) LODs, it was possible to abolish this approach and to certify the non-GMO CRM applying the qPCR method directly on the ground non-GMO powder and certifying it to have a GMO content below the LOD of the method applied.

RMPs in general aim to produce and certify a CRM which allows maintaining the traceability chain to a common reference. The problem for GMO CRMs is that what is measured (the DNA copy number ratio) has no fixed relation to what is intended to be measured (the GMO mass fraction). This relationship between DNA copy number ratio and GMO mass fraction varies between seed batches and is impacted by biotic and abiotic factors of the growing conditions, as well as storage conditions and time. Furthermore, there are several different seed lines commercialized and planted per GMO event. While all GMO CRMs are certified in mass fractions with a measurement unit traceable to the kilogramme of the International System of Units (SI), the variation of the biological product means that replacement sets of GMO CRMs can have the same GMO mass fraction but are likely to differ in their DNA copy number ratio. It was attempted to minimize/eliminate this variation by ensuring that the DNA content of the GMO and non-GMO powders does not differ from each other significantly [17]. For this, the DNA content was measured with a slightly modified classical fractionation method [18]. After sequential removal of alcohol-, alcohol-ether-, and acid-soluble compounds, the DNA was extracted and its mass measured in a spectrophotometer after a colorimetric reaction [19]. Commercial DNA extraction kits, aiming at PCR amplifiable DNA, could not be employed as they extract varying fractions of the DNA. With the classical fractionation method, different DNA contents were observed for different seed lots. These were at least partially caused by the differing growing conditions for the non-GMO and GMO seeds, which per default were grown well isolated from each other, and which often also led to visually different average seed sizes. Likewise, earlier GMO CRMs systematically used the near isogenic line of GMO event as non-GMO counterpart. The near isogenic line, however, was sometimes not commercialized and either only produced in very small quantities or under completely different growing conditions. Consequently, it is impossible to ensure that different seed batches have exactly the same DNA content and hence the same DNA mass fraction. This means that all mixtures of GMO powders setting up a calibration curve should come from the same GMO CRM batch.

Ultimately, this led to the understanding that the GMO CRM together with EURL GMFF validated method sets up the reference system for EU legislation. One consequence of this is that CRM users have to start a new quality control sheet when changing from one set of GMO CRMs to the next [20]. The certified values on a GMO CRM certificate have a validity. In contrast to other CRMs, the RM user shall not extend the validity of certificate, disrespectful of possible confirmatory measurement results, if the CRM set has been replaced by the RMP by a new one. CRM replacement sets are at JRC identified by the small letters k, l, m, n, o, p, etc. following the CRM code (e.g. as in ERM-BF413k).

## Commutability

Together with EU’s GMO reference system for GMO quantification, the understanding about GMO CRMs and their commutability evolved. The official GMO CRMs are prepared from pure non-GMO and GMO seed powders to implement the mass fraction-based thresholds set in the corresponding EU legislation for food and feed. The seed matrix is chosen as being close to food/feed products checked for labelling compliance. The CRMs are intended for quality control and calibration of the qPCR method validated by the EURL GMFF. As only one validated method and one official GMO CRM set up the reference system for quantification in the EU, no specific commutability assessment needs to be carried out and due to the restriction to one (reference) method also no commutability assessment can be carried out. If GMO CRMs are used which are not the official GMO CRMs, the CRM user needs to demonstrate that they lead to the same measurement result (with the EURL GMFF validated qPCR method), for correct implementation of thresholds stipulated in EU legislation. The same is true if other GMO quantification methods are used: the users need to demonstrate equivalence of the result.

The first GMO CRMs were produced from seeds. The request for sugar beet and later on potato GMO CRMs allowed to apply alternative approaches for the purity testing. In contrast to seeds, beets and tubers are larger, meaning that only a few of them will be needed for CRM production. Testing all tubers or beets entering the processing individually allowed to certify them as 0 and 100% GMO with negligible or even zero uncertainty [21, 22]. In the case of sugar beets, the beets were peeled; for potatoes, this was not done, as most DNA is contained in the peel and as starch processing industry also uses unpeeled potatoes for the extraction of starch. While differences in size and hence the surface (peel) to volume ratio between GMO and non-GMO products could further impact the differences between the copy number ratio measured by qPCR and the certified mass fraction, the approach to peel all potatoes, freeze-dry peel and flesh separately and generate mixtures of the same peel to flesh ratio was consider but finally not followed as it would have resulted in non-representative materials.

## Plasmid DNA for calibration of DNA copy number ratios

In theory, biotech companies could commission several RMPs to produce GMO CRMs for the same event. One of the produced CRMs would be part of the authorization process and hence ‘official’. For the other, ‘unofficial’ CRM, the user needs to demonstrate that it leads to the same measurement result. As this is work intensive, the motivation to produce additional CRMs is very limited, leading to one set of official CRMs per GMO event. As pointed out earlier, this restriction entails that no independent materials can be used for quality control and for calibration and that quality assurance can only partially be carried out by the analytical laboratories.

In 2004, EC recommended expressing the results of GMO measurements as DNA copy number ratio [9]. EC JRC therefore invested in the development of plasmid DNA CRMs [23, 24], intended for calibration of the qPCR method while the powder-based CRMs, certified for their GMO mass fractions, were intended for quality control.

The plasmids contain two fragments, one of the junction region and one of the endogenous species gene targeted by the EURL GMFF validated qPCR method. These CRMs for calibration are buffer solutions containing the plasmids and background DNA. They are certified for the number of fragments per plasmid and an indicative value for the ratio measured by qPCR is provided. Four calibrants containing plasmid DNA (pDNA) were developed and released (see Table 2: ERM-AD413, ERM-AD415, ERM-AD425, ERM-AD427). The advantage of this approach is that the calibration with a plasmid is completely independent from the matrix CRM used for quality control.

However, with the implementation of (EU) No 619/2011, it was clarified that GMO measurement results shall be expressed in mass fractions, which as a consequence means that measurement results obtained in copy number need to be transformed into mass fractions using the official GMO CRM as reference. Therefore, the need for a certified pDNA material disappeared, laboratories wishing to work with an in-house calibrant, can do so, but are obliged to link the value of their calibrant to the official GMO CRM.

## GMO CRM releases and dissemination

The legal requirement to have a GMO CRM released at the issuing of EFSA’s scientific opinion requires the availability of the CRM long before a GMO event is possibly authorized for the EU market. The RMP may gain a monopoly for 10 years (following publication of the implementation decision) for the GMO CRMs of a specific event but is also required to maintain a CRM, which is not yet applied routinely. Furthermore, changed ownership or commercialization strategies can lead to withdrawal of the authorization request and an unsecure recovery of the investment cost made by the RMP. GMO CRM productions are only undertaken by JRC if the intention of the GMO company is to seek market authorization of their event under (EC) No 1829/2003. Evaluation of this retrospectively shows that more than one-third of the released set of GMO CRMs are not used for the implementation of (EC) No 1829/2003. Reasons are diverse and range from changed authorization intentions (food use vs industrial use), retraction of the authorization request or not prolonging an expiring authorization. It also includes pending authorization requests (see Table 2). Nevertheless, the fact that a specific GMO CRM is only made available by one RMP allows an interesting evaluation of the demand and geographical distribution of the CRM users.

**Fig. 3:**
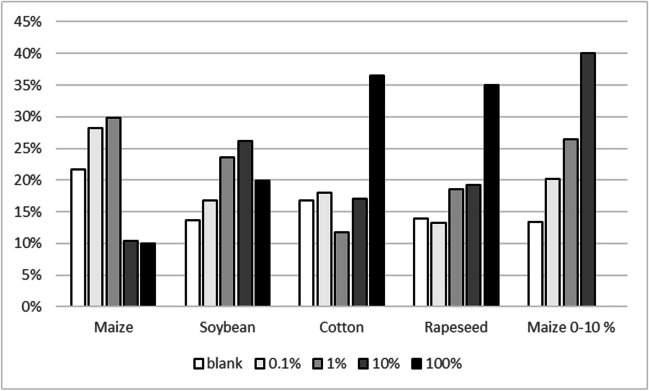
Distribution of individual GMO CRM units sold per mass fraction levels. Average sales of CRM units from sets with nominal 0, 0.1, 1, 10, and 100% (m/m) compared to sets with nominal 0, 0.1, 1, and 10% (m/m)

**Fig. 4:**
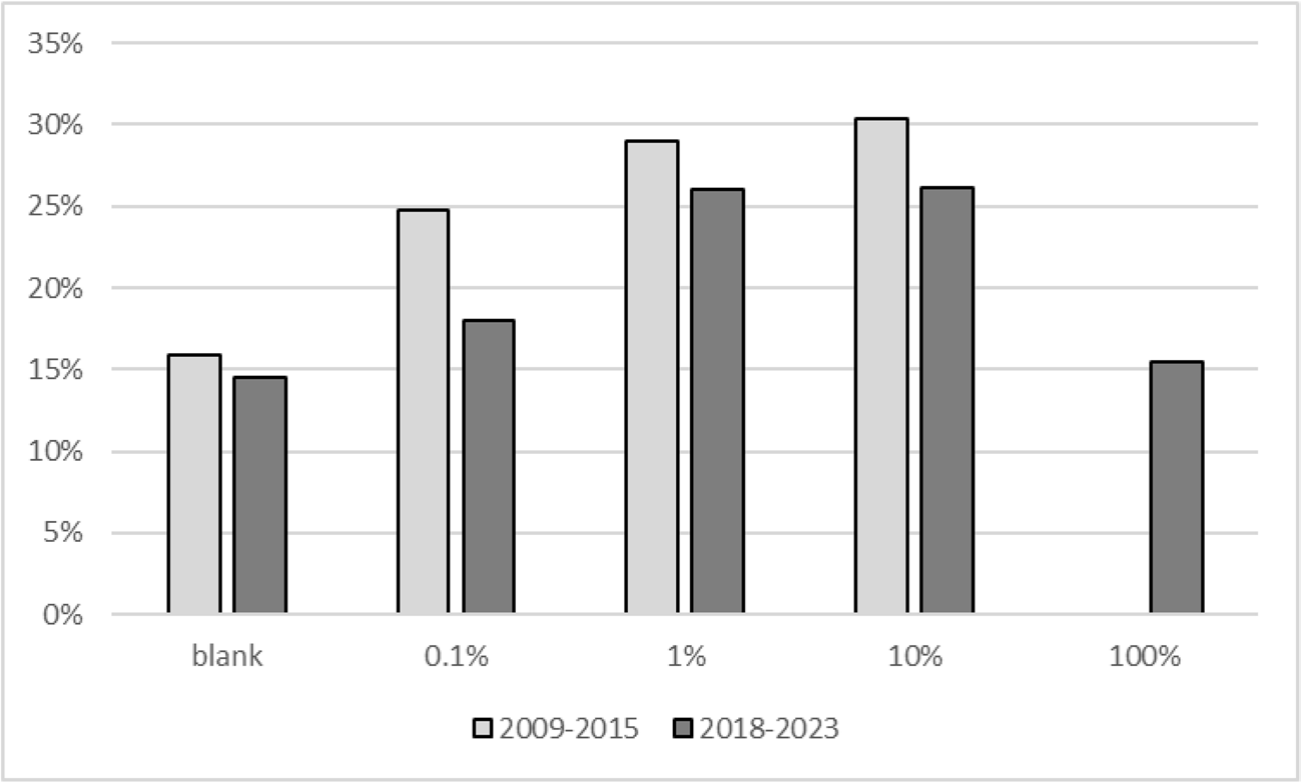
Distribution of individual units sold per mass fraction levels of GTS 40-3-2 soybean GMO CRMs (set of ERM-BF410 CRMs). Between 2009 and 2019, only nominal 0 to 10 % mass fractions were available; in 2018 a new set including a nominal 100 % mass fraction was released

Figure 3 shows the distribution of the number of sold CRM units for materials where sets of nominal 0, 0.1, 1, 10, and 100% mass fractions are available for the four genetically modified species for which JRC produces GMO CRMs. No clear pattern emerges here: For cotton and rapeseed CRMs, the 100% material is the most requested, whereas this is not the case for maize or soybean. This can probably be explained by the fact that cotton seeds and rapeseeds are used for oil production in which DNA-based measurements are challenging. Interesting is the comparison with maize CRMs where only 0, 0.1, 1, and 10% mass fractions and no 100% mass fraction material was made available. The 10% material largely seems to substitute the 100% material, but availability of a 100% material seems to have an impact on all mass fractions except the nominal 0%. This might be because customers prepare their own mixtures using DNA extracted from the nominal 0% and 100% material. A similar picture emerges for the CRMs for GTS 40-3-2 soybean (ERM-BF410). In the beginning, only nominal 0, 0.1, 1, and 10% were available (years 2009 to 2015). After an intermediate period with some bridging CRMs, a new set of CRMs consisting of nominal 0, 0.1, 1, 10, and 100% became available in 2018. Also here the 100% material partially substitutes for all other mass fractions (see Fig. 4).

**Fig. 5:**
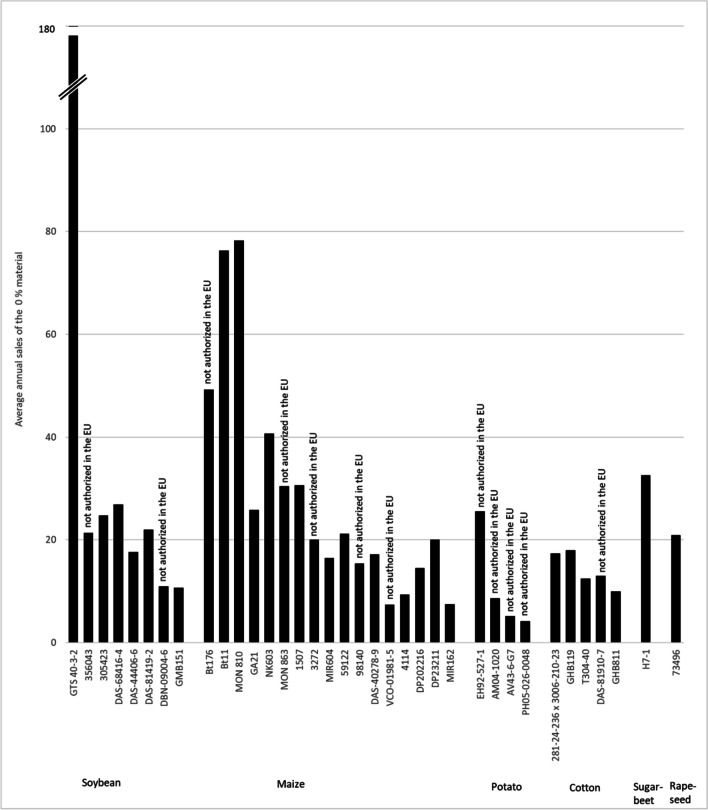
Average annual sales of the nominal 0% (m/m) GMO CRMs in the years 2009 to 2022. Note the break in the *y*-axis for GTS 40-3-2 soybean. Average annual sales for GTS 40-3-2 (ERM-BF410a) is 177 units. The EU authorization status in 2024 under (EC) No 1829/2003 is given. DP202216 and DP23211 were authorized in 2024, given sales numbers therefore represent the pre-authorization phase

**Fig. 6:**
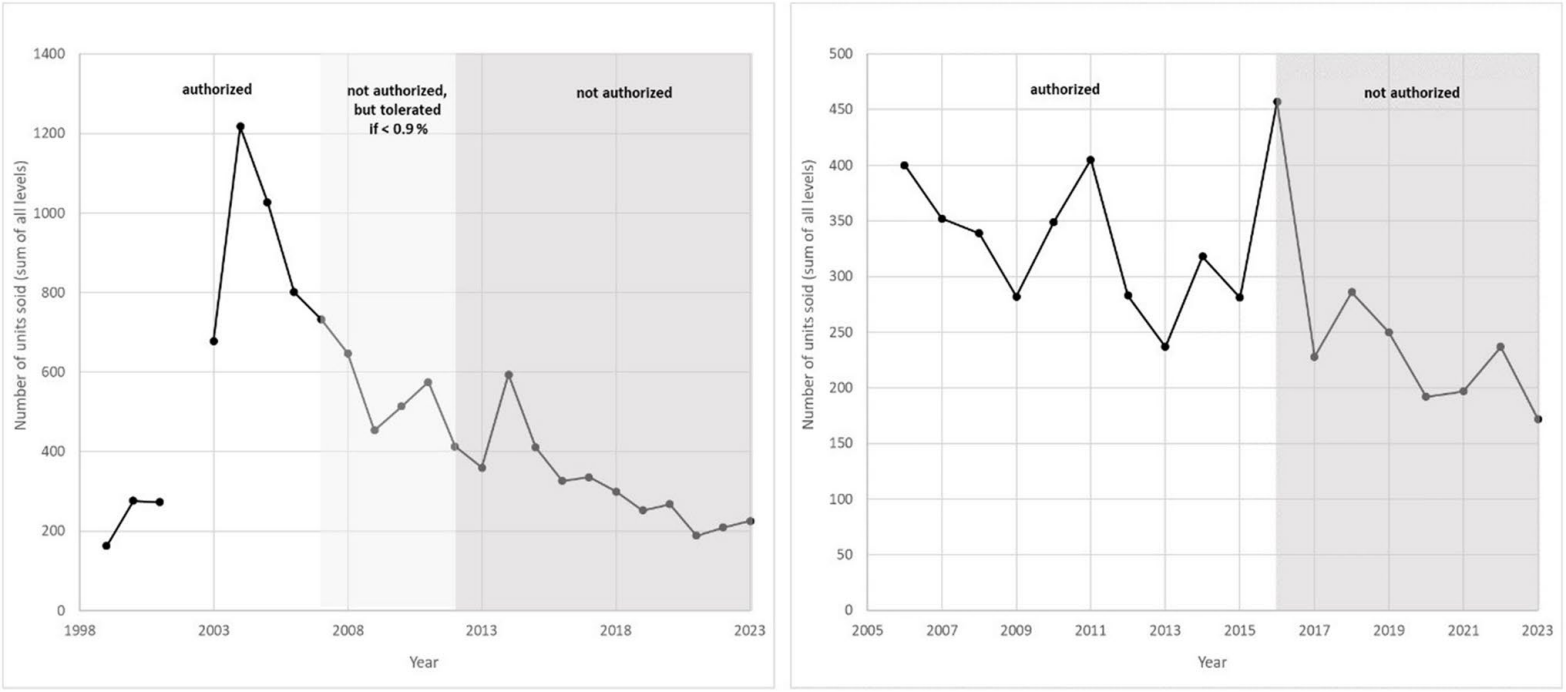
Sales figure of CRMs for Bt176 maize (ERM-BF411) (left) and MON 863 maize (ERM-BF414) (right) with indication of the EU authorization status of the events. Note that ERM-BF411 was withdrawn in 2002 and a new material was only released in 2003

The intensity of use for the various CRMs varies widely. Figure 5 shows the average annual sales of the nominal 0% GMO CRMs. The 0% material was chosen as it is available for all CRMs and is hardly affected by which other mass fractions are available (see above). By far, the most widely used are the CRMs for GTS 40-3-2 soybean, followed by MON 810, Bt11, and Bt176 maize. These were also the first CRMs released by the JRC and the first GMO events marketed commercially. The demand for CRMs is not obviously correlated with the authorization status of the events. As seen in Fig. 5, the demand for CRMs seems to be to a large extent independent from the authorization status. The CRMs for Bt176 maize (ERM-BF411) and MON 863 maize (ERM-BF416) allow a deeper investigation of this effect. ERM-BF411 was released in 1998. The authorization was withdrawn in 2007 with a period of 5 years where the presence of the GMO maize was tolerated as long as it was below 0.9%. The sales figures (Fig. 6) show a clear increase for the Bt176 maize CRMs when (EC) No 1829/2003 came into force. However, after this sharp peak, the demand declines and there is no indication of a steeper decline after the withdrawal of the authorization or the end of the grace period. The same is true for the MON 863 CRMs, where the decline of the yearly sales after withdrawal of the authorization matches the trend of the years before. It therefore seems that the authorization status of an event is not a useful predictor of the demand for the corresponding CRMs.

**Fig. 7:**
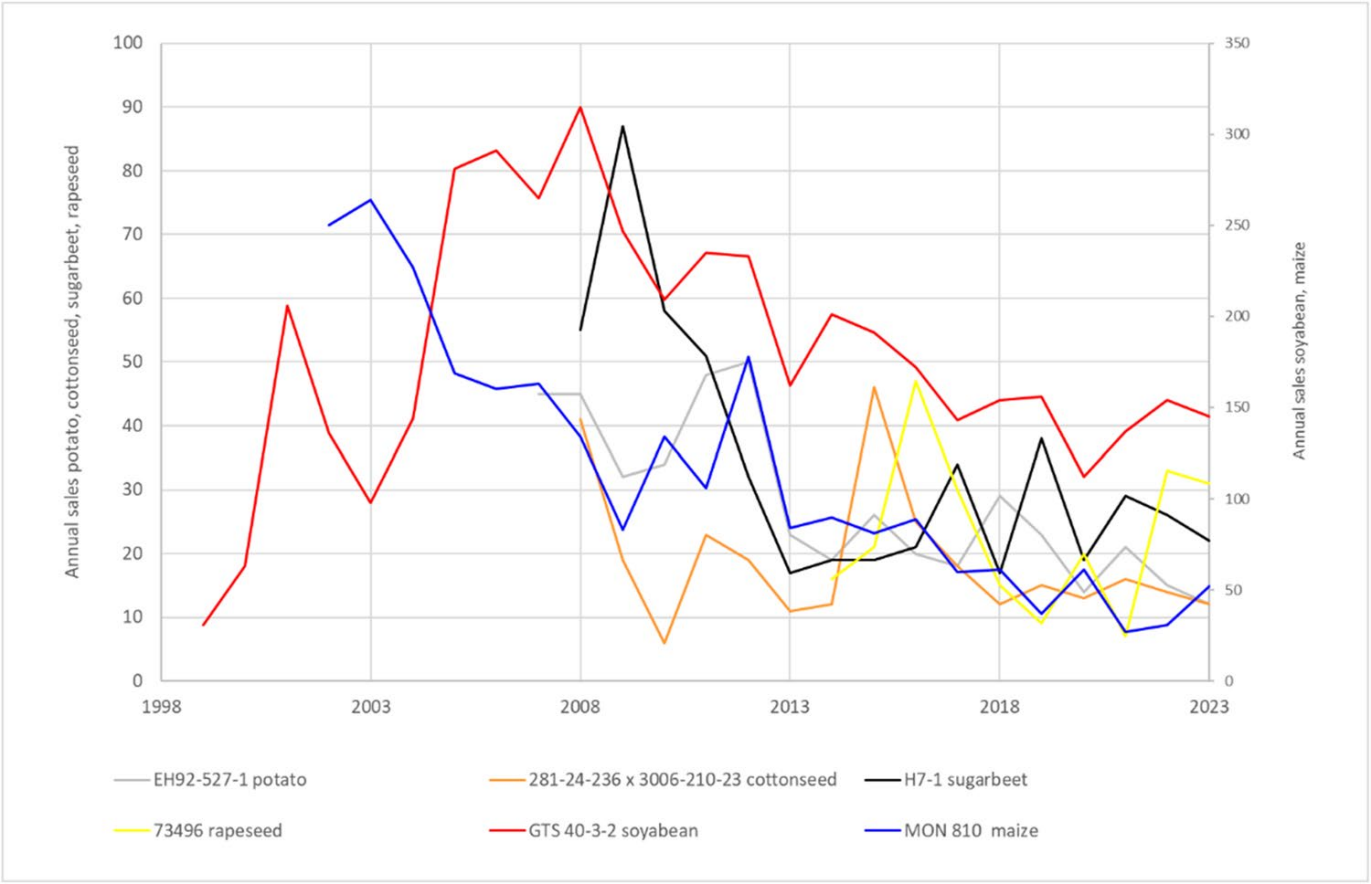
Number of CRM units sold of the nominal 0% (m/m) materials since 2009. Note that the sales figures for the GTS 40-3-2 soybean and MON 810 maize are on the secondary vertical axis

The sales of the GMO materials shows an overall declining trend since 2009 (see Fig. 7). The dip in the year 2020 might be due to the COVID-19 pandemic: several laboratories contacted for the stability monitoring analyses refused to be commissioned as their qPCR machines were used for COVID-19 testing. However, similar dips also happened before (e.g. 2013) without any clear explanation.

Another interesting aspect is the demand for DNA copy number certified GMO CRMs. Between 2007 and 2011, JRC released four plasmid CRMs, intended for the calibration of DNA copy number measurements by qPCR (ERM-AD413, ERM-AD415, ERM-AD425, and ERM-AD427). The nominal 2% mass fraction of the MON 810 maize seed powder-based (matrix) CRM (ERM-BF413ek) is additionally certified for its DNA copy number ratio. As Regulation (EU) No 619/2011 in 2011 explicitly specified and therefore clarified that GMO measurements results should be given in mass fraction %, the plasmid CRMs became less relevant.

**Fig. 8:**
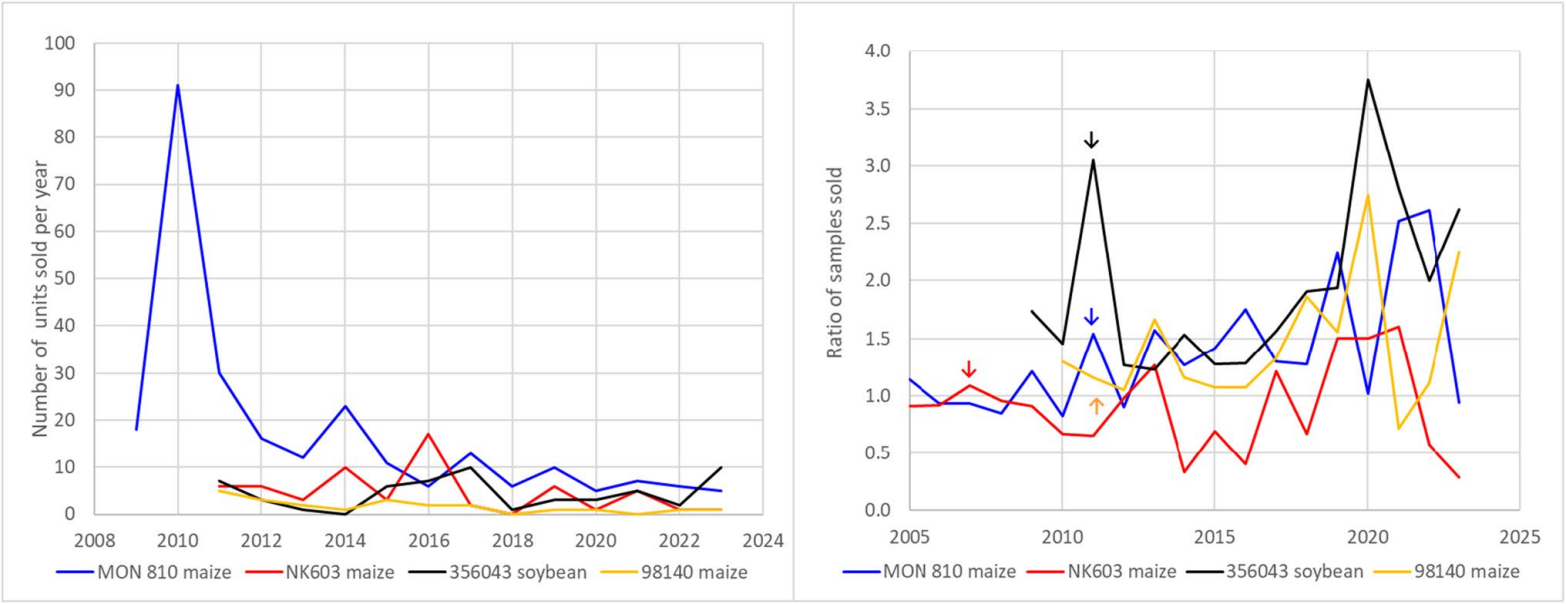
Sales of copy number GMO CRMs. Left: Sales figures for plasmid DNA CRMs, originally intended for the calibration of qPCR measurements over time. Right: Ratio of sales of CRMs certified for their DNA copy number concentration over the respective 0% CRMs. The arrows indicate the year when the additional certified value for the copy number concentration was added

**Fig. 9:**
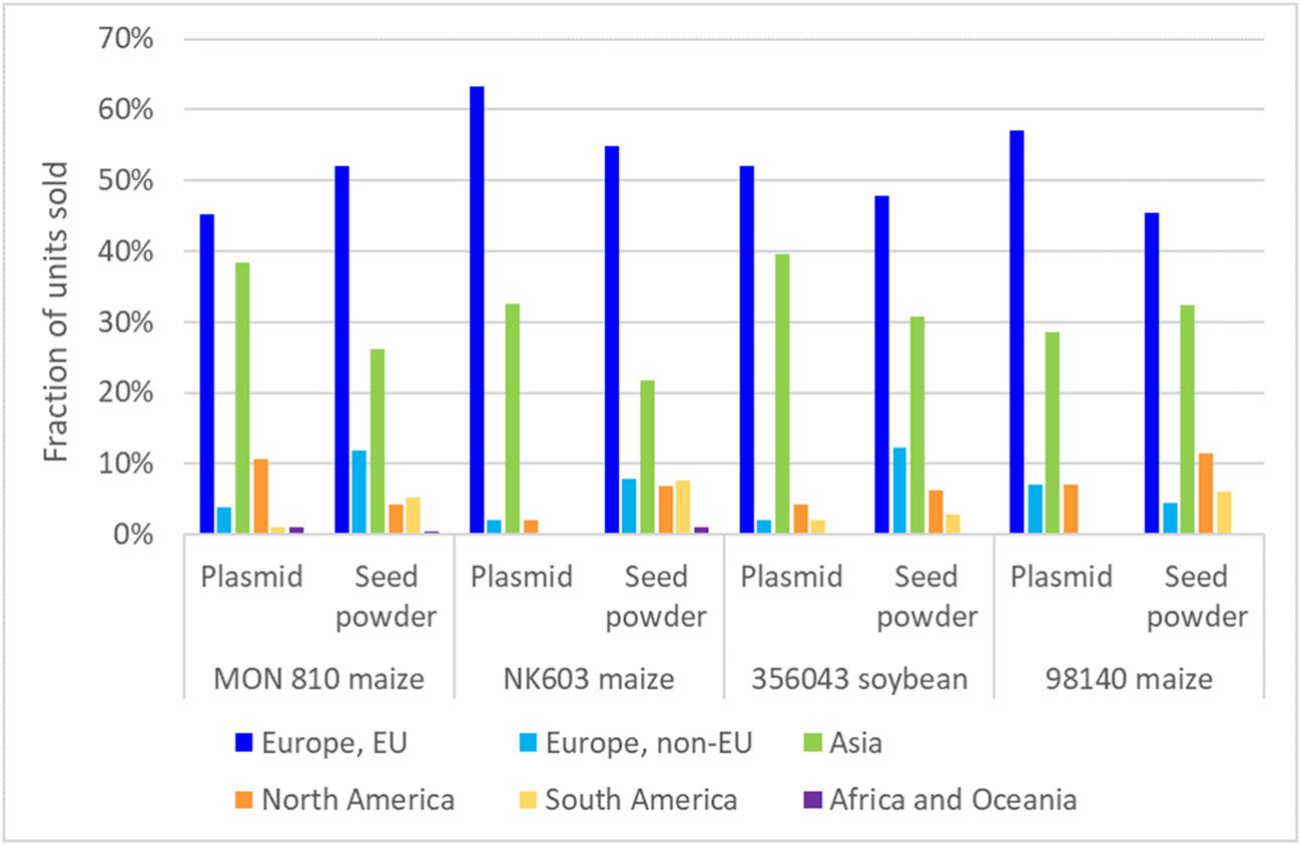
Geographical distribution of the sales of plasmid CRMs and the corresponding powder CRMs. Values are percentages of the sales since 2013

Figure 8 (left) shows the development of the sales of the four plasmid CRMs over time. The material for MON 810 maize (ERM-AD413) shows the rapid increase, which is common for materials after introduction to the market, but could as well be caused by the fact that JRC certified a MON 810 maize material for its DNA copy number ratio and its GMO mass fraction (ERM-BF413ek), which allowed a direct comparison of the different measurement results in dependence of the calibration curve setup. After a high in 2010, sales decreased, most likely because of the specification that the percentages of the regulation are understood as mass fraction percentage making them appear less relevant. It is interesting to note that even if Regulation (EU) No 619/2011 stipulates reporting in mass percentages, there is still a small demand for plasmids. Figure 9 compares the geographical distribution of the sale of these plasmid CRMs with those of the corresponding powder CRMs. Again, no clear pattern emerges with the dominant markets being the EU and Asia. Since 2013, GMO plasmid CRMs were sold to a total of 31 different countries. The certification of the copy number ratios in the powder materials requires significant resources and the price of CRMs with additional certified values for their copy number ratios is therefore 60% higher than the price of CRMs only certified for their mass fractions. Figure 8 (right) shows the ratio of the annual sales of CRMs certified also for their copy number compared to the corresponding blank material. For all materials, there is neither a clear increase in demand upon the release of the certified value for the copy number ratio, nor is there a clear decline after (EU) No 619/2011 specified that results need to be given in mass fractions and hence rendering this additional value less useful. The production and development of GMO CRMs at JRC were a reaction to EU legislation, but the distribution of the CRMs is by far not restricted to the EU.

**Fig. 10:**
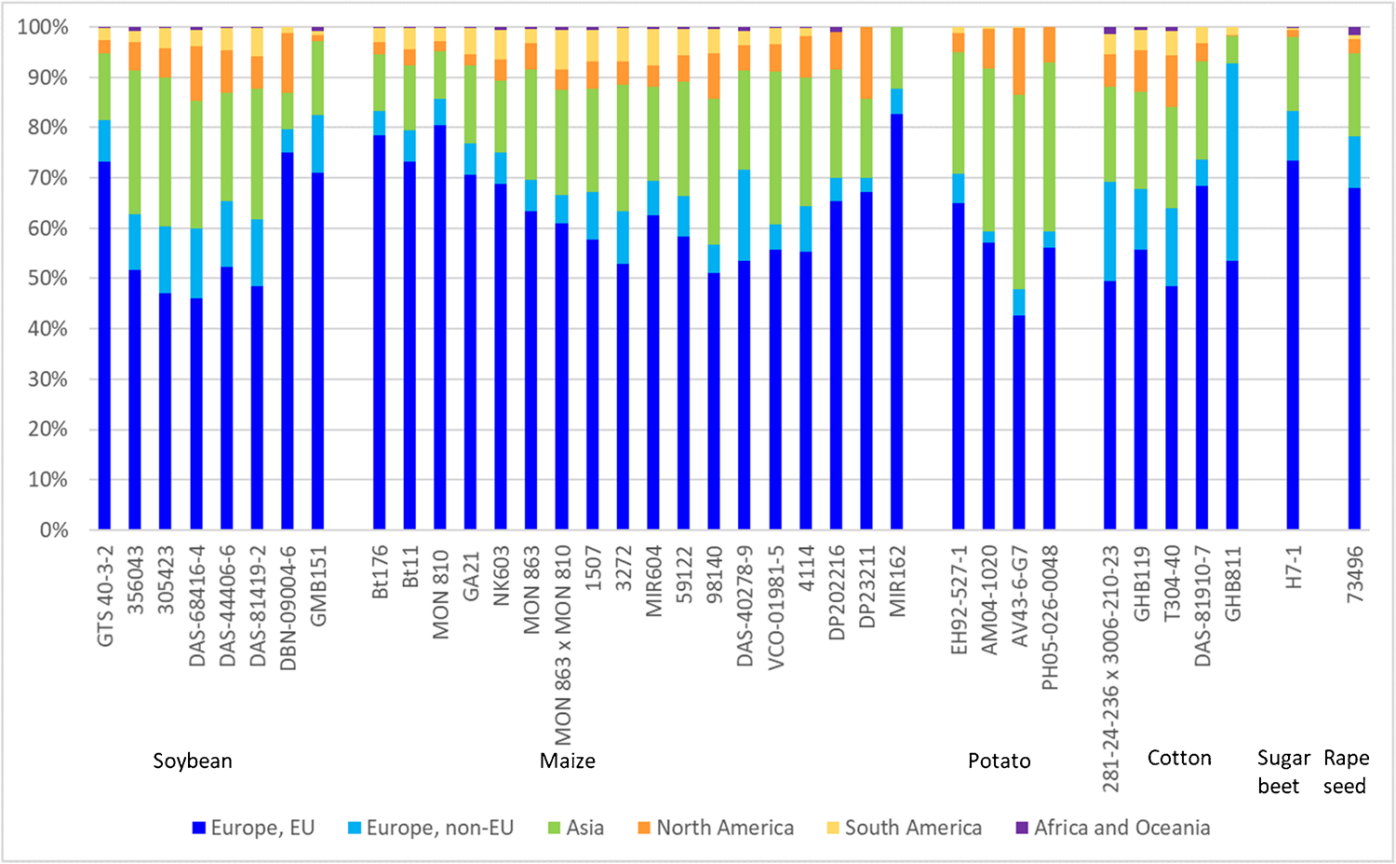
Destination of JRC’s GMO CRMs during the years 2009 to 2022. Relative sales numbers per region are given

Figure 10 shows the geographical distribution of the sale of JRC’s GMO CRMs and the relative percentage of the overall sales of all GMO CRMs. As the need for GMO CRMs was triggered by EU regulation, it is not surprising that the majority of CRMs is sold to customers in Europe, mainly the EU. The second biggest destination is Asia in this group, 26% of all GMO CRMs going to the People’s Republic of China, 20% to Japan, 12% to the Republic of Korea, 11% to Thailand, and 7% to the Republic of China (Taiwan). Given the wide acceptance of GMOs in the USA and Canada, the low use of these CRMs in these countries is expected. South America, especially Brazil, is the major destination in the Americas, which is not astonishing seen that Brazil is the biggest country in South America and grew already in 2018 more than a quarter of the GMO crops worldwide [25]. The distribution of sales of GMO CRMs per region is fairly stable over time.

Unfortunately, it is not possible to distinguish between groups of customers (e.g. official control laboratories, contract laboratories, research organizations). The reason is that a considerable fraction of CRM units are sold via re-sellers and no information on their final customers is available to the JRC. In addition, in several cases, contract laboratories also act as official control laboratories, making this distinction impossible.

## JRC’s strategy related to GMO CRMs

RM development and production require the investment of resources. Being part of the European Commission, the JRC generally focuses on CRMs which support the implementation of EU legislation and which are not offered by other RMPs. The JRC has its own intervention logic that puts the support to European policies and priorities in its focus. As a non-profit organization, the JRC avoids to enter in a competitive scenario when high-quality RMs that are fit for purpose are offered by other RMPs.

Related to GMO CRMs, an evaluation carried out in 2016 [26] confirmed that neither the Commission nor the EU Reference Laboratory needs to produce these reference materials as EU legislation does not specify the RMP. This means that the JRC does not hold exclusive rights on this type of RMs. However, so far, only one other RMP has taken up the production of GMO CRMs [27] and offers to biotech companies to produce the GMO CRMs required for EU market authorization. In principle, also other RMPs could offer this type of RMs, given that the materials have the required level of quality and the RMP holds an accreditation scope for this type of RMs.

In 2021, an impact case study of JRC’s RMs confirmed the support delivered by JRC with respect to official food control laboratories in the EU who are responsible for implementing GMO legislation. At that time, the share of GMO soybean products being used in a global market was found to be worth USD 128 billion in 2019 and an expected USD 146 billion by 2025 [28]. The feedback of users on the quality of JRC’s CRMs is generally very positive and is expressed bilaterally or in frequently organized customer satisfaction surveys.

The increased experience at the JRC led to the reduction of the development time for a set of GMO CRM to 14 months. While the development time for a GMO CRM is in comparison to other matrix RMs short, the waiting time for the GMO event to be authorized is longer, in which the demand for the CRM is generally low. Likewise, changes of ownership of a GMO event or changed market strategies can lead to the retraction of the application by the biotech company, leading to resource investments by the RMP which are likely not to be recovered.

The Joint Research Centre, as a part of the European Commission, provides independent, evidence-based science and knowledge, supporting EU policies to positively impact society. This means that its scientific priorities must also follow the general political priorities of the European Commission. These priorities change over time and require adjustments of the scientific topics for research and development, as numerous new challenges ask for a redistribution of resources and planned work. As described above, the JRC is not the sole producer of GMO CRMs and the market is open for potential future RMs provided by other RMPs. Current and future developments in NGTs could result in scenarios where traditional GMO RMs would not be needed anymore. However, this depends on the future shape of regulations with regard to NGTs and its details, such as the categorization of NGT plants into those that could also naturally occur or as a result of conventional breeding and all other NGT plants. The latter would fall under the current GMO legislation, requiring an authorization procedure. This political discussion is still ongoing in the EU and the outcome of this process cannot be predicted. Having all these considerations in mind, the JRC is considering to end the development of new GMO RMs, but will in any case continue to provide existing GMO RMs for many years to come. Any new demands for RMs made for organisms developed by NGTs require reviewing the current CRM production approach for its suitability and efficiency and re-assessing the situation.

## Conclusions

GMO CRM development, production, and distribution are essential for the implementation of current EU legislation on GMOs. The official GMO CRM and the EURL GMFF validated qPCR method set up the reference system for the measurements and ensure a common EU GMO labelling thresholds. EC JRC has produced since more than 25 years GMO CRMs and distributed them predominantly to laboratories in the EU. Over the years, the production of GMO CRMs has been optimized, which ensured production efficiency and suitability of the CRM for the implementation of EU legislation on GMOs.

However, the need to have a GMO CRM released for the scientific opinion in the authorization process requires the availability of the CRM long before a GMO event is possibly authorized for the EU market. While biotech companies seek an early release of the GMO CRM, RMPs get no guarantee that the CRM produced will become the official CRM. Changed ownership of the GM events or changed commercialization strategies during this lag phase can lead to withdrawal of the authorization request, a low usage of the CRM, and an unsecure recovery of the investments made by the RMP. Adjusting the legal framework could reduce this lag phase, could reduce unnecessary investments, and could also help other RMPs to enter into this area.

Upcoming dPCR methods require the establishment of a common conversion factor to transform copy number values measured by calibration-free dPCR into mass fraction values as currently requested by EU legislation. As a conversion factor is already published by the EURL GMFF for the highest concentrated GMO CRM of each set of CRMs, the need to produce different GMO mass fractions’ levels per event should be re-evaluated. A reduction to produce and release only a pure GMO (nominal 100%) material could be justified.

Additionally, it should be considered that the current labelling thresholds of 0.9% and 0.1% are established by the reference system established by official GMO CRM and the validated EURL GMFF method. Seeing its arbitrary nature, it could be considered to change to DNA copy number-based thresholds, which can directly be measured by dPCR and do not require mass fraction certified GMO CRMs for calibration. However, such a change would require a transparent communication to the EU citizens to explain the difference between the more tangible mass of GMOs and the technically directly measurable DNA copy numbers of GMOs.

## Outlook

New genomic techniques (NGTs) raise analytical challenges to discriminate between plants developed with NGTs from plant variants produced by conventional (including mutation) breeding or modified by natural mutations. Analytical techniques such as targeted or whole genome sequencing may be increasingly applied and rather require access to DNA sequence databases. Current EU legislation on GMO authorization requires GMO CRMs for implementing a common GMO labelling threshold for food and feed products [29]. Continued needs for GMO CRMs will therefore to a large extent depend on whether future EU legislation will require authorized NGT products to be labelled above a defined threshold.
